# Communicating multiple tubular enteric duplication with toxic megacolon in an infant: A case report

**DOI:** 10.1097/MD.0000000000025772

**Published:** 2021-05-07

**Authors:** Eunju Jang, Jae Hee Chung

**Affiliations:** Department of Surgery, Seoul St. Mary's Hospital, The Catholic University of Korea, Seoul, Korea.

**Keywords:** case report, gastrointestinal tract duplication, infant, surgery, toxic megacolon

## Abstract

**Rationale:**

Gastrointestinal tract duplication is a rare congenial anomaly which can be found anywhere along the gastrointestinal tract. While many patients are incidentally diagnosed during operation, in some cases it can present with severe gastrointestinal symptoms. In this case report, the patient presented with signs of toxic megacolon leading to rapid aggravation of inflammatory shock.

**Patient concerns:**

A 49-day old male infant presented with fever, poor feeding, and severe abdominal distension.

**Diagnosis:**

Abdominal ultrasonography was done. During the examination, a foley catheter was inserted through the anus to evaluate bowel patency and enable rectal decompression. The tip of the foley catheter was located in a separate narrower tubular lumen adjacent to the distended rectum. These findings suggested possibility of a tubular duplication cyst of the rectum as the culprit for the bowel obstruction.

**Interventions:**

The patient underwent emergency laparotomy. Findings showed multiple tubular intestinal duplications involving the ileum, appendix, cecum, descending colon, sigmoid colon and rectum. The true lumen of the rectosigmoid colon was completely collapsed while the adjacent tubular cyst remained severely distended and stool passage was not possible. Decompression of the sigmoid colon was done with loop colostomy with both the wall of the true bowel and enteric cyst forming the colostomy orifice.

**Outcomes:**

After 40 days of postoperative care, the patient was discharged with no immediate complications. Four months after the initial operation, colostomy take-down and transanal rectal common wall division was done. No complications were observed.

**Lessons:**

To our knowledge, this is the first case to be reported where a rare presentation of intestinal duplication resulted in an acute presentation toxic megacolon. Such emergency cases can be effectively treated with emergency surgical bowel decompression and elective common wall division.

## 1 Introduction

Gastrointestinal tract duplication is a rare congenial anomaly, with an incidence of 1 in 4500 live births.^[1]^ It can be found anywhere along the gastrointestinal tract; most commonly in the ileum (33%), followed by the esophagus (20%), colon (13%), jejunum (10%), stomach (7%), and duodenum.^[2]^ As the locations vary, enteric duplication presents with a wide spectrum of symptoms.^[3]^ Duplications are structurally divided into cystic (86%) and tubular types (14%), and the cystic lesion usually does not communicate with the lumen of the adjacent bowel and contains a sticky mucoid fluid. Tubular lesions often have one or more direct communications with the adjacent bowel and can appear either as double-barreled or Y-shaped forms.^[4]^ Because many patients are asymptomatic and clinical presentations frequently overlap with other gastrointestinal abnormalities, most enteric duplications are diagnosed incidentally during surgery.^[5]^

Here we present a rare case of an infant who presented with shock due to toxic megacolon caused by noncontinuous multiple enteric tubular duplications with communication from terminal ileum to rectum. To our knowledge, this is the first case to be reported where a rare presentation of intestinal duplication resulted in an acute presentation of inflammatory shock caused by toxic megacolon.

The caregivers of the patient included in this case provided informed consent at the time of surgery to be included in this case report.

## 2 Case presentation

**Figure 1:**
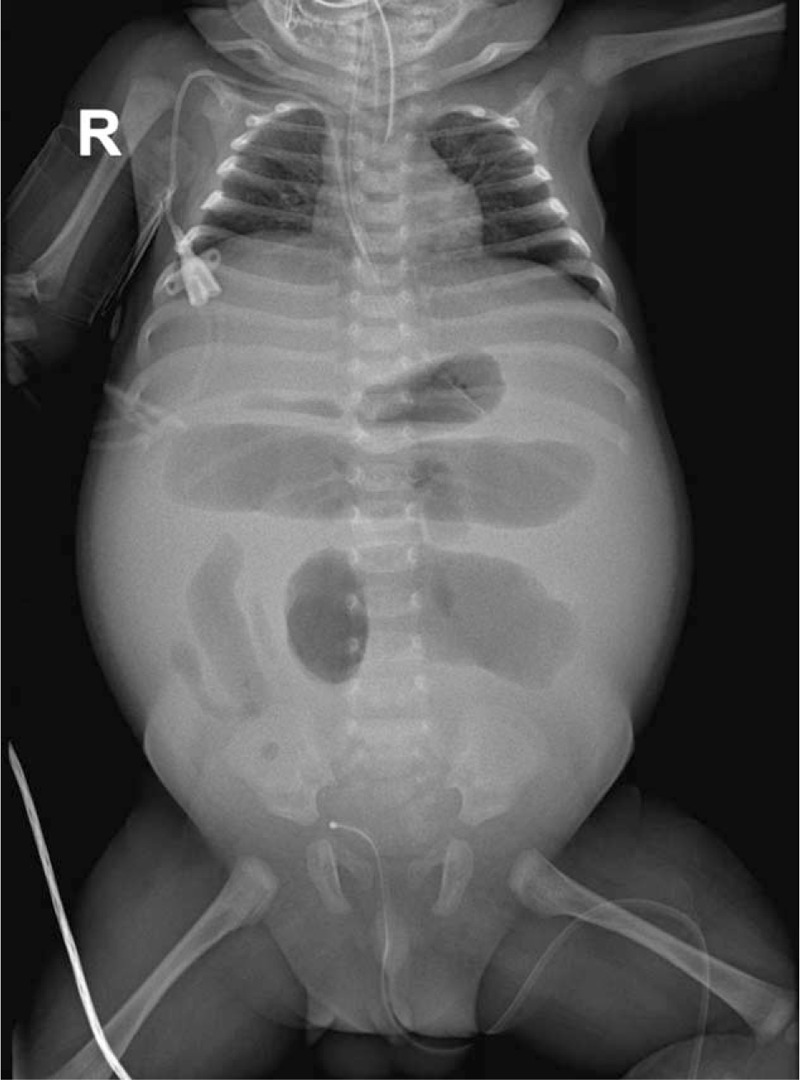
Initial plain radiography on PICU admission. Abdomen was distended with colon wall dilatation to approximately 5 cm with bowel edema.

A 49-day old male baby presented to the emergency department with fever, poor feeding, and abdominal distension which started 1 day ago. Initial lab findings showed CRP elevation, leukocytosis, and bowel distension on plain x-ray, suggesting possible congenital megacolon (Fig. 1). He was admitted for treatment of enteritis and evaluation for megacolon including colon study. He had a previous history of neonatal intensive care unit admission after birth due to neonatal pneumonia and had received ventilator care. Congenital pharynx deformity was observed for which resection of pharyngeal polyp was done and also congenital ptosis of the right eye and mild sacral deformity was suspected. During neonatal intensive care unit care, the infant showed signs of repeated colon distension and colon study was done. However, other than mildly decreased bowel motility of the transverse megacolon, no specific findings were apparent and eventually normal defecation was observed. Oral feeding was gradually increased and 27 days after admission, the patient was discharged.

**Figure 2:**
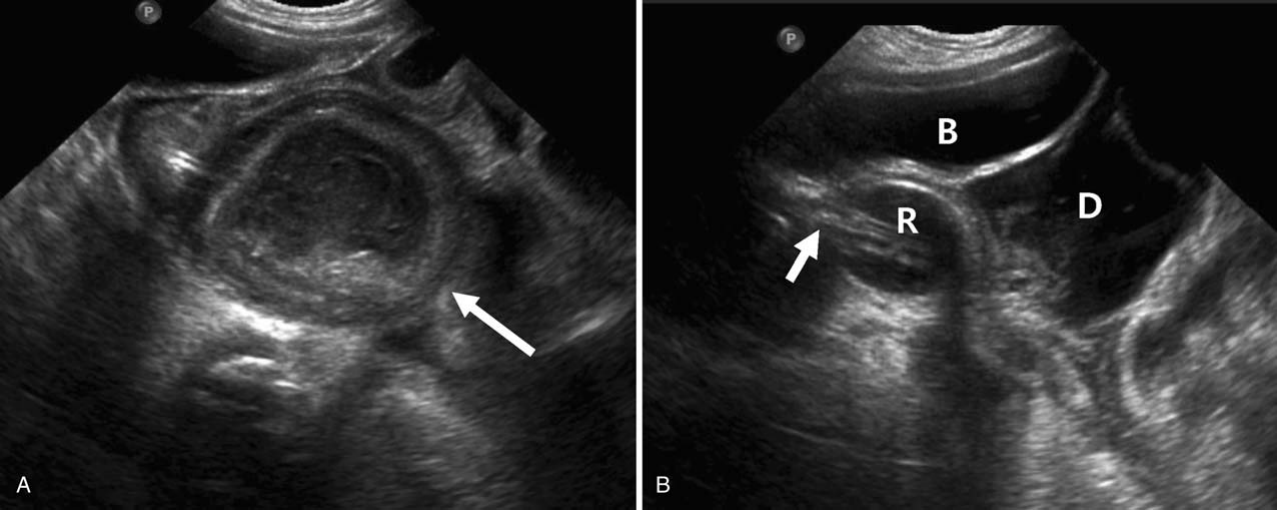
(A) Ultrasound showed bowel wall thickening with extremely distended colon and rectum, which indicated toxic megacolon. (B) Ultrasound after foley insertion through the anus. The balloon tip of the foley catheter passed through the true rectum adjacent to the distended duplication bowel. (Arrow: foley catheter, B: bladder, D: duplication cyst, R: rectum).

However, few days after discharge, the amount of oral feeding decreased to less than half, and the infant had extreme difficulty in defecating. Although rectal tube insertion was immediately done, decompression was ineffective and abdominal distension aggravated with worsening signs of shock. Patient was admitted to PICU where resuscitation with hydration, inotropics, mechanical ventilation, and combined antibiotic therapy with vancomycin, meropenem, and metronidazole was initiated. Emergency ultrasonography findings showed clear signs of toxic megacolon with marked distension of rectum and distal colon, diffuse bowel wall thickening and turbid fluid collection (Fig. 2A). During ultrasonography, foley catheter was inserted through the anus to enable simultaneous rectal decompression. However, the tip of the foley catheter was located in a separate narrower tubular lumen adjacent to the distended rectum, which explained the ineffectiveness of rectal drainage (Fig. 2B). These findings were suggestive of tubular duplication cyst of the rectum as a possible cause of toxic megacolon.

**Figure 3:**
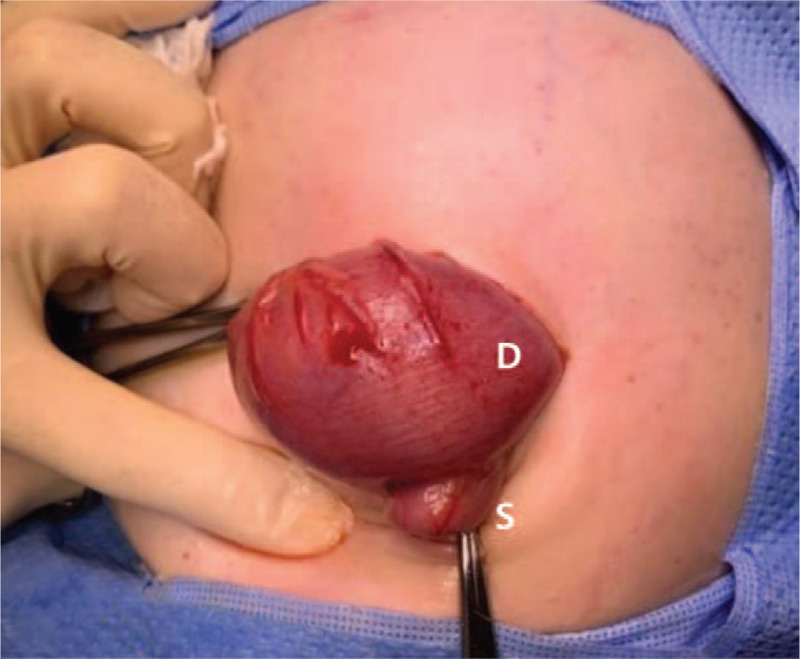
Duplication in the sigmoid colon. Duplicated cyst showed severe distension (D), while the true lumen (S) was collapsed.

**Figure 4:**
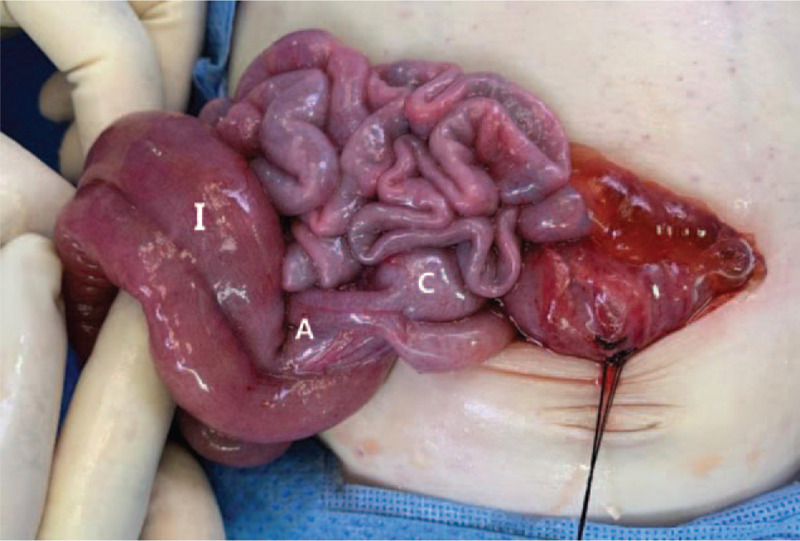
Operation findings showed multiple duplication cyst involving the terminal ileum (I), appendix (A), and cecum (C).

After initial resuscitation, emergency laparotomy was done. Our suspicion for enteric duplication was confirmed, with operative findings showing multiple tubular intestinal duplication involving distal ileum, appendix, cecum, descending colon, sigmoid colon, and rectum (Figs. 3 and 4). One extended from the splenic flexure colon to the distal rectum with a proximal communication and a blind end, and a separate duplication started from the distal ileum to the cecum with a duplicated appendix. Both ends of the duplication communicated with the true bowel lumen. The true lumen of the rectosigmoid colon which contained the rectal tube inserted preoperatively, was completely collapsed while the adjacent tubular cyst was severely distended and stool passage was not possible. Decompression of the sigmoid colon with loop colostomy was done with the true bowel and enteric cyst both forming the colostomy orifice.

Postoperative ICU care was required for management of shock and bowel inflammation. Continuation of antibiotics, mechanical ventilation, intravenous inotropic, electrolyte replacement, hydration, and hyperalimentation was done. Steroid therapy was also applied as inflammation persisted after negative culture results. After 2 weeks of steroid therapy, symptoms and laboratory results showed marked improvement. The patient was stable after discontinuation of inotropic, mechanical ventilation, and normal defecation via colostomy was observed. The patient was transferred to the general ward and oral feeding was tolerable. After 40 days of postoperative care, the patient was discharged with no immediate complications.

Four months after the initial operation, colostomy take-down, and transanal rectal common wall division was done using a surgical stapler (endo-GIA^TM^, Coviden) in order to make a single lumen between the true and duplicated rectum. The patient was discharged 1 week after the operation in healthy condition. Follow-up examination showed good prognosis and no complications such as anal stricture or constipation were observed.

## 3 Discussion

Most enteric duplications are diagnosed prenatally, while symptomatic patients present with various symptoms such as abdominal pain, bilious emesis, respiratory symptoms, abdominal mass, constipation, and incidental intraoperative findings.^[6]^ Structurally, spherical cysts are most common (83%–91%) compared to tubular cysts (8%–16%). Duplications typically do not communicate with the adjacent lumen, and share a common wall with the gastrointestinal tract (71%) but some cysts have a luminal communication with the bowel (37%).^[1,4]^ Multiple duplication cysts are rather uncommon (1%–7%) and multiple duplication cysts involving multiple segments of the GI tract, such as our patient are even less common than multiple enteric cysts involving a single segment.^[7]^

Operative findings showed multiple communicating tubular cysts involving multiple regions of the gastrointestinal tract. Although septic shock was initially suspected, no bacteria or any microorganisms were detected in blood, stool, ascites or genitourinary culture which was routinely repeated every 3 days during ICU care. We assume systemic inflammatory response syndrome caused by endotoxemia resulted in shock. It can be suspected that stool accumulation in the tubular duplication cyst involving the sigmoid colon and rectum progressed over the 49 days after birth. This would have caused the severe dilation of the double-barreled enteric cyst to protrude into the true bowel lumen, causing mechanical colon obstruction. Tubular duplication involving the ileum and cecum communicated with no signs of mechanical obstruction and both lumens were approximately the same size.

Cases of enteric duplications associated with life-threatening sepsis have been reported, in which intestinal ischemia and post-operative septic shock led to high mortality. Two neonates with midgut volvulus caused by congenital duplication cyst underwent operation in which extensive bowel resection was inevitable due to gangrenous changes to long segments of the small bowel.^[5]^ A different case of isolated infection of duplication cyst has also been reported, clinical presentation showed signs of mild colitis, and elective cyst excision was done after antibiotic therapy.^[8]^

Toxic megacolon is quite rare in pediatric patients, but there were reported cases where toxic megacolon due to Hirshprung disease have been managed surgically.^[9,10]^ These patients underwent emergency operation which involved extensive resection of the distended colon. Surgical colonic decompression with fecal diversion alone was reported to be associated with higher complication rate and mortality in treating toxic megacolon due to Hirshprung disease.^[10]^ To the best of our knowledge, our case is the first in literature describing toxic megacolon associated with congenital enteric duplication. In our experience, decompression with colostomy followed by intensive medical care was effective in toxic megacolon. This allowed elective resection of the common wall and colostomy take-down after complete recovery from shock.

In terms of surgical treatment of enteric duplication cysts, complete resection of the duplication cyst is the treatment of choice, due to risk of recurrent attacks of gastrointestinal symptoms or malignant changes.^[11]^ Elective resection using minimally invasive surgery have shown good outcomes.^[2,11]^ However, in case of long tubular duplication of the colon, subtotal or total colectomy is sometimes inevitable due to a common blood supply with the native bowel. Also, duplications involving the rectum may require the resection of rectum which could lead to undesirable results in a pediatric patient. Division of the common wall in an enteric duplication has previously been performed as a simpler and safer alternative to extensive colonic resection.^[12]^ In our patient, diagnostic laparotomy for management of toxic megacolon was done and after full recovery, elective resection of the common wall was done with endo-GIA through the transanal approach, minimizing the extent of bowel resection and risk of surgery-related complications.

## 4 Conclusion

This is a first reported case of a multiple tubular enteric duplication accompanied with toxic megacolon. Temporary loop colostomy and delayed resection of the common wall was done to avoid extensive bowel resection. Decompression with colostomy was effective in treatment of toxic megacolon, and surgical management of duplication of cysts with minimal resection showed good postoperative results. In pediatric patients with atypical clinical presentations, possibility of a congenital defect must always be considered. Sonographic exams combined with physical examinations can be effective in making a diagnosis. Emergency operation to manage toxic megacolon, and delayed operation to correct the bowel deformity proved efficient and effective.

## Author contributions

**Conceptualization:** Jae Hee Chung.

**Data curation:** Eunju Jang.

**Formal analysis:** Eunju Jang.

**Investigation:** Eunju Jang, Jae Hee Chung.

**Supervision:** Jae Hee Chung.

**Validation:** Jae Hee Chung.

**Writing – original draft:** Eunju Jang.

**Writing – review & editing:** Eunju Jang.
